# Within-host population dynamics of extensively drug-resistant *Mycobacterium tuberculosis* revealed by an over 3-year longitudinal study

**DOI:** 10.1093/emph/eoaf014

**Published:** 2025-07-01

**Authors:** Peng Xu, Xuecong Zhang, Chi Wu, Yiwang Chen, Wenjie Lai, Liyong Liu, Jialei Liang, Dan Li, Ruimin Hong, Senlin Zhan, Peize Zhang, Howard Takiff, Guofang Deng, Jiuxin Qu, Qian Gao

**Affiliations:** National Clinical Research Center for Infectious Diseases, Shenzhen Clinical Research Center for Tuberculosis, Shenzhen Third People’s Hospital, Shenzhen, China; National Clinical Research Center for Infectious Diseases, Shenzhen Clinical Research Center for Tuberculosis, Shenzhen Third People’s Hospital, Shenzhen, China; Department of Clinical Laboratory, Shenzhen Third People's Hospital, The Second Affiliated Hospital, School of Medicine, Southern University of Science and Technology, Shenzhen, China; Key Laboratory of Medical Molecular Virology (MOE/NHC/CAMS), School of Basic Medical Science, Shanghai Medical College, Shanghai Institute of Infectious Disease and Biosecurity, Fudan University, Shanghai, China; Department of Clinical Laboratory, Shenzhen Third People's Hospital, The Second Affiliated Hospital, School of Medicine, Southern University of Science and Technology, Shenzhen, China; Department of Clinical Laboratory, Shenzhen Third People's Hospital, The Second Affiliated Hospital, School of Medicine, Southern University of Science and Technology, Shenzhen, China; Key Laboratory of Medical Molecular Virology (MOE/NHC/CAMS), School of Basic Medical Science, Shanghai Medical College, Shanghai Institute of Infectious Disease and Biosecurity, Fudan University, Shanghai, China; National Clinical Research Center for Infectious Diseases, Shenzhen Clinical Research Center for Tuberculosis, Shenzhen Third People’s Hospital, Shenzhen, China; National Clinical Research Center for Infectious Diseases, Shenzhen Clinical Research Center for Tuberculosis, Shenzhen Third People’s Hospital, Shenzhen, China; Department of Pulmonary Medicine and Tuberculosis, Shenzhen Third People’s Hospital, Southern University of Science and Technology, Shenzhen, China; Department of Pulmonary Medicine and Tuberculosis, Shenzhen Third People’s Hospital, Southern University of Science and Technology, Shenzhen, China; Laboratorio de Genética Molecular, CMBC, Instituto Venezolano de Investigaciones Científicas, IVIC, Caracas, Venezuela; National Clinical Research Center for Infectious Diseases, Shenzhen Clinical Research Center for Tuberculosis, Shenzhen Third People’s Hospital, Shenzhen, China; Department of Pulmonary Medicine and Tuberculosis, Shenzhen Third People’s Hospital, Southern University of Science and Technology, Shenzhen, China; National Clinical Research Center for Infectious Diseases, Shenzhen Clinical Research Center for Tuberculosis, Shenzhen Third People’s Hospital, Shenzhen, China; Department of Clinical Laboratory, Shenzhen Third People's Hospital, The Second Affiliated Hospital, School of Medicine, Southern University of Science and Technology, Shenzhen, China; National Clinical Research Center for Infectious Diseases, Shenzhen Clinical Research Center for Tuberculosis, Shenzhen Third People’s Hospital, Shenzhen, China; Key Laboratory of Medical Molecular Virology (MOE/NHC/CAMS), School of Basic Medical Science, Shanghai Medical College, Shanghai Institute of Infectious Disease and Biosecurity, Fudan University, Shanghai, China

**Keywords:** tuberculosis, extensively drug-resistant TB, microevolution, ineffective treatment, drug resistance mutation

## Abstract

**Background and objectives:**

Drug resistance is a major contributor to tuberculosis (TB) deaths worldwide. Understanding the dynamics of in-host evolution of *Mycobacterium tuberculosis* (MTB) drug resistance can help to improve treatment success rates.

**Methodology:**

The microevolution of drug-resistant MTB was studied in three patients with long-standing, extensively drug-resistant TB (XDR-TB) by analyzing whole genome sequences of serial isolates collected during treatment.

**Results:**

We identified three patterns of *in vivo* MTB microevolution during long-term, ineffective treatment: (i) new drug-resistant subpopulations emerge and compete with other subpopulations during treatment; (ii) drug resistance profiles remaining stable without the emergence of new drug-resistant subpopulations; and (iii) after a drug is stopped, new drug-resistant subpopulations continue to emerge and compete with existing subpopulations.

**Conclusions and implications:**

The microevolution of drug-resistant MTB within patients on long-term ineffective treatment is complex. Subpopulations with different resistance-conferring mutations can compete with each other and with newly emerged subpopulations. Often, one subpopulation eventually dominates and achieves long-term stability. This work deepens the understanding of MTB microevolution in XDR-TB patients.

## INTRODUCTION

Tuberculosis (TB) is, since the waning of the COVID-19 pandemic, again the leading cause of death globally from a single infectious agent [1], averaging nearly 1.5 million deaths per year over the past decade [2]. Drug-resistant TB is a major cause of TB treatment failure [3], with a treatment success rate of just 68% in patients with rifampicin-resistant (RR-TB) or rifampicin and isoniazid resistant (multidrug-resistant or MDR) TB [2]. TB strains that are MDR/RR and also resistant to a fluoroquinolone and either bedaquiline or linezolid (or both), are termed extensively drug-resistant TB (XDR-TB) [4] and have even higher rates of treatment failure [5], often leading to fatal outcomes.

Unlike other bacteria that can acquire drug-resistant genes through plasmids and horizontal gene transfer, the development of drug resistance in *Mycobacterium tuberculosis* (MTB) is caused solely by mutations in the MTB genome [6], which are acquired through a process of microevolution under selection pressure from antibiotics [7]. There are currently two principal models for MTB microevolution *in vivo*. In the first, MTB are exposed to selective drug pressures but the mutations conferring resistance also carry fitness costs [8], leading to subpopulation competition and fixation of the mutations that balance the least fitness costs with the highest levels of resistance [9, 10]. In the second model, TB patients have multiple lesions, and the MTB in different lesions have their own, independent microevolutionary trajectories [7], leading to the long-term coexistence of multiple subpopulations within TB patients. Both models may occur simultaneously in patients, and the process of microevolution of drug-resistant subpopulations in patients may be even more complicated due to the diverse environments and immunological defense mechanisms within the host. Understanding the microevolution of drug resistance in MTB can help to optimize treatment strategies, especially for difficult strains of TB that are resistant to several anti-TB drugs.

Although one study has documented the evolution of a drug susceptible strain into XDR-TB over a 42-month period [11], most studies on the within-host evolution of drug resistance are based on short-term observation periods [7, 12]. As a result, the microevolution of MTB drug resistance in patients who receive prolonged but ineffective treatments remains unclear. In this study, we enrolled three patients with long-standing XDR-TB and collected their serial isolates obtained during drug treatment to trace the microevolution of their MTB populations.

## METHODOLOGY

### Patient enrolment and isolate preparation

The study included three patients with drug-resistant TB who were diagnosed and treated at Shenzhen Third People’s Hospital between 2018 and 2024 and had more than 10 isolates obtained during treatment. Information on patients’ treatment regimens, imaging, and medication adherence was collected anonymously. All preserved MTB isolates were resuscitated from frozen stocks grown on Löwenstein–Jensen (L-J) medium at 37°C for 3 weeks. Colonies were scraped from the surface of the L-J slope and their genomic DNA was extracted with the Cetyltrimethylammonium Bromide (CTAB) method.

### Analysis of whole genome sequencing data

Whole genome sequencing (WGS) of MTB genomic DNA was generated with 150 bp pair-end reads on the Illumina NovaSeq X Plus sequencing platform by Novogene Co. Ltd (China). Following a previously described analysis pipeline [9], Single Nucleotide Polymorphisms (SNPs) were identified by excluding repetitive regions of the genome, including the proline–glutamic acid (PE) and proline–proline–glutamic acid (PPE) gene families, phage sequences, insertions, and mobile genetic elements. To exclude potential sequencing errors, each identified SNP was supported by a depth of at least 5×, a frequency of at least 5%, and at least one read in each direction. Frequencies ≥95% were defined as fixed SNPs whereas frequencies <95% were defined as unfixed SNPs. False positives for unfixed SNPs were excluded based on their frequency differences using the protocol of Liu *et al*. [13]. Subpopulations were defined as strains carrying unfixed SNPs, and the frequency of a SNP was defined as the proportion of total sequencing reads containing that variant. A mixed infection is when two or more genetically distinct strains of MTB coexist in the host [14]. Mixinfect, a Bayesian model-based clustering analysis method, was used to identify mixed infections with ≥10 heterozygous sites and Bayesian Information Criterion (BIC) values >20. The lineages of the MTB isolates were determined using lineage-specific SNPs identified by Napier *et al*. [15].

### Genotypic and phenotypic drug susceptibility testing

Genotypic drug susceptibility was predicted using SAM-TB (v2.0) [16] and the phenotypic drug susceptibility was determined using the minimum inhibitory concentration (MIC) test. For first- and second-line anti-TB drugs [rifampicin (RIF), isoniazid (INH), ethambutol (EMB), moxifloxacin (MFX), ofloxacin (OFX), amikacin (AMI), kanamycin (KAN), streptomycin (STR), ethionamide (ETO), para-aminosalicylic acid (PAS), and cycloserine (CS)], the MIC was determined using the Sensititre MYCOTB plate (ThermoFisher Scientific, USA). For new/repurposed anti-TB drugs [bedaquiline (BDQ), clofazimine (CFZ), delamanid (DLM), and linezolid (LZD)], the MIC was determined using the MTB MIC kits (Baso Diagnostics, Inc., China). Phenotypic resistance was defined using the critical concentrations specified by the WHO: RIF > 0.5 μg/ml; INH > 0.12 μg/ml; EMB > 4.0 μg/ml; MFX > 0.5 μg/ml; OFX > 2.0 μg/ml; AMI > 1.0 μg/ml; KAN > 5.0 μg/ml; STR > 2.0 μg/ml; ETO > 5.0 μg/ml; PAS > 2.0 μg/ml; CS > 32 μg/ml; BDQ > 0.5 μg/ml; CFZ > 0.5 μg/ml; DLM > 0.5 μg/ml; and LZD > 0.5 μg/ml [17].

Strains were classified as drug resistant based mainly on the phenotypic drug susceptibility testing (DST) results. Strains carrying mutations associated with resistance in the WHO catalogue [18] but phenotypically susceptible were also classified as drug resistant. Phenotypic DST was not performed for pyrazinamide (PZA) and capreomycin (CAP), and genotypic DST results were used to determine drug susceptibility for these drugs. Effective drugs were defined as those to which the strain was susceptible. Treatment regimens containing at least four drugs to which the isolates were susceptible were defined as effective treatment, whereas regimens with fewer than four drugs to which the isolates were susceptible, were defined as ineffective treatment [19].

## RESULTS

### Patient and serial isolates

Three TB patients (A, B, and C) were included in this study. They were all men, aged 52, 41, and 42 years who had been treated with anti-TB therapy for 45, 74, and 60 months, respectively. In total, 12, 20, and 11 clinical MTB isolates, respectively were obtained from patients A, B, and C. All isolates from the three patients belonged to lineage 2 (Beijing lineage). Self-discontinuation and irregular dosing were noted in all three patients during the course of their treatments, and patient C was unable to take ETO and PZA due to severe liver toxicity. Computerized tomography (CT) showed that each patient had 4–6 cavities in the lungs, with lesions in all lobes.

To assess the genetic diversity of MTB within patients, we evaluated the WGS data to find changes in fixed and unfixed SNPs in the serial isolates from each patient. The isolates from each patient contained a maximum difference of seven fixed SNPs (Supplementary Fig. S1), with the majority differing by three or fewer fixed SNPs. No mixed infections or exogenous reinfections were detected. Compared to the first isolate obtained from each patient, none of the subsequent isolates contained more than nine new unfixed SNPs, and 85% of the isolates carried ≤5 unfixed SNPs. Of all the unfixed SNPs identified, 76% had frequencies below 25%. Patients A, B, and C had 7, 5, and 0 unfixed SNPs, respectively, that became fixed SNPs in subsequent isolates, (Supplementary Fig. S2). Thus, despite the slow mutation rate for MTB and the overall stability, the genomes continued to evolve, introducing diversity into the in-host MTB population while exposed to ineffective treatment regimens.

### Genotypic and phenotypic DST results

**Figure 1:**
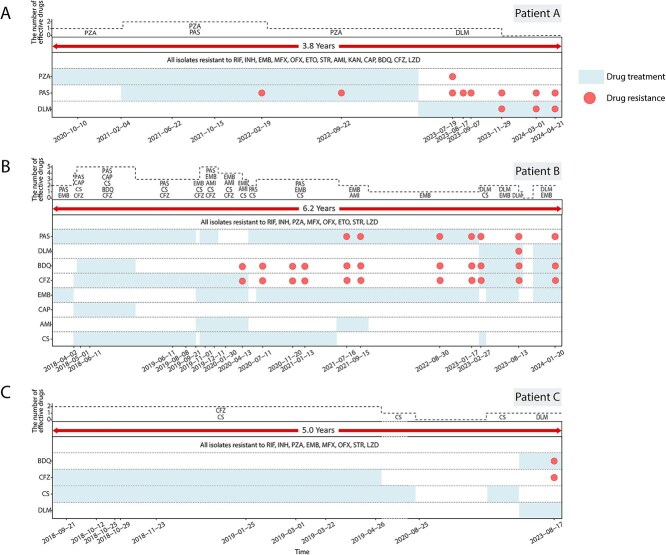
Time-panel of clinical treatment and drug resistance acquisition. The composite figure shows the treatment regimen, strain resistance, and the number of effective drugs (dashed lines) for patients A, B, and C. The blue bars indicate when the patient was receiving each drug. At the bottom of each panel, the time points indicate when an isolate was collected from the patient. Abbreviations: RIF, rifampicin; INH, isoniazid; EMB, ethambutol; PZA, pyrazinamide; PAS, para-aminosalicylic acid; MFX, moxifloxacin; OFX, ofloxacin; ETO, ethionamide; AMI, amikacin; KAN, kanamycin; CPA, capreomycin; STR, streptomycin; CS, cycloserine; BDQ, bedaquiline; CFZ, clofazimine; DLM, delamanid; LZD, linezolid.

The genotypic and phenotypic DST predictions for resistance to the first- and second-line anti-TB drugs were identical except for ETO and STR (Supplementary Table S1). At the time of enrollment into the study, the three patients were already resistant to most first- and second-line anti-TB drugs (Fig. 1). Because of the low accuracy for genotypic prediction of resistance to new/repurposed anti-TB drugs [18], phenotypic DST was used for predicting resistance to BDQ, CFZ, DLM, and LZD. This study showed that, at the time of enrollment, patient A was resistant to BDQ, CFZ, and LZD, while patients B and C were resistant to LZD. During subsequent treatment, patient A developed resistance to DLM, patient B to BDQ, CFZ, and DLM, and patient C to BDQ and CFZ (Fig. 1). We analyzed the concordance between genotypic and phenotypic DST results for these four drugs and found that drug resistance mutations (DRMs) were detected in 45.8% (11/24) of isolates that were phenotypically resistant to BDQ, in 45.8% (11/24) phenotypically resistant to CFZ, in 50.0% (1/2) phenotypically resistant to DLM and in 100% (43/43) of isolates phenotypically resistant to LZD (Supplementary Table S1).

### Patterns of MTB microevolution within patients

**Figure 2:**
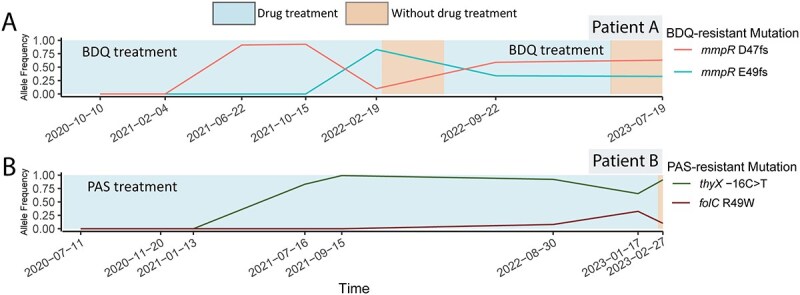
Emergence of new drug-resistant subpopulations and competition among subpopulations during treatment. The line graphs indicate changes in the frequencies of resistant subpopulations, the emergence of new resistant subpopulations, and the competition between subpopulations. Blue areas indicate when the patient was receiving the medication and orange areas indicate when the patient was not receiving the medication. Panel A shows the frequencies of two BDQ-resistant subpopulations carrying the *mmpR* D47fs and *E49fs* mutation, respectively, in serial isolates from patient A, taken during treatment with BDQ on the dates indicated at the bottom. Panel B shows the change in the frequencies of two PAS-resistant subpopulations carrying mutations in the *folC* and *thyX* genes, respectively, in serial isolates from patient B, during treatment with PAS. Abbreviations: BDQ, bedaquiline; PAS, para-aminosalicylic acid.

**Figure 3:**
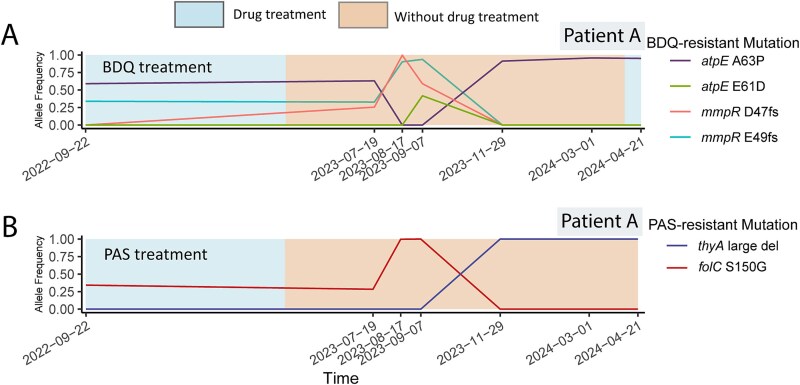
New resistant subpopulations in patient A emerge and existing subpopulations compete after the drug is removed. Line graphs show the frequencies of resistant subpopulations, the emergence of new resistant subpopulations and competition between subpopulations. Blue areas are when the patient received the indicated drug and orange areas indicate when the patient did not receive the drug. Panel A shows changes in the frequencies of BDQ-resistant subpopulations in the serial isolates from patient A, taken at the times shown on the bottom. The subpopulation carrying the *atpE* E61D mutation (green line) emerged after BDQ was stopped. Panel B shows changes in frequencies of PAS-resistant subpopulations in serial isolates from patient A. The subpopulation carrying the *thyX* gene deletion (blue line) emerged after PAS was stopped. Abbreviations: BDQ, bedaquiline; PAS, para-aminosalicylic acid.

The treatment regimen has a significant impact on the microevolution of resistant MTB [19]. We analyzed the patients’ treatment regimens and the resistance profiles of their isolates and found that patient B had been prescribed effective treatment for only 15 months (15/74), whereas patients A and C had been given only ineffective treatment regimens (Fig. 1). By analyzing the dynamics of DRMs in the MTB isolates from the three patients on long-term ineffective treatment, we identified three microevolutionary patterns:

(i) New drug-resistant subpopulations emerge and compete during treatment. The phenotypic DST showed that all three patients developed new resistant subpopulations during drug treatment (Fig. 1). While receiving BDQ, the MTB population within patient A successively generated two competing BDQ-resistant subpopulations that alternately dominated in the population, each with a different frameshifting (fs) mutation in the *mmpR* gene: one subpopulation carried the D47fs mutation (arising after 10 months of BDQ treatment); the other carried the E49fs mutation (arising after 18 months of BDQ treatment; Fig. 2A). The ratio of the D47fs subpopulation to the E49fs subpopulation changed from 1:8 on 19 February 2022 to 2:1 on 22 September 2022 (Fig. 2A). Similarly, patient B, while receiving treatment with PAS, developed two competing PAS-resistant subpopulations: one with a mutation in *thyX* which remained dominant in the population; the other with a mutation in *folC* (Fig. 2B). The ratio of the *thyX* mutation subpopulation to the *folC* mutation subpopulation changed from 12:1 on 30 August 2022, to 2:1 on 17 January 2023, to 9:1 on 27 February 2023 (Fig. 2B).
(ii) During long-term ineffective treatment, the resistance profile remains stable with no additional drug resistance detected by either genotypic or phenotypic DST. Patient B did not develop resistance to several antibiotics despite receiving them during long-term treatments: EMB (taken for 48 months); CS (39 months); AMI (12 months); and CAP (9 months; Fig. 1B). Patient C was given CS and DLM for 24 and 9 months, respectively, without developing resistance to either drug (Fig. 1C).
(iii) New resistant subpopulations emerge after the drug is stopped. Patient A developed a BDQ-resistant subpopulation (carrying the *atpE* E61D mutation) after stopping BDQ for 5 months (Fig. 3A), and a PAS-resistant subpopulation (carrying a large deletion in *thyA*) after stopping PAS for 7 months (Fig. 3B). After patient A stopped receiving BDQ, competition continued between several BDQ-resistant subpopulations carrying different mutations, with the subpopulation carrying the *mmpR* D47fs mutation eventually becoming fixed while the other subpopulations became undetectable (Fig. 3A). Similarly, after patient A stopped receiving PAS, the subpopulation with a deletion in *thyA* emerged and eventually became fixed, while the previously dominant subpopulation carrying the *folC* S150G mutation eventually became undetectable (Fig. 3B).

## DISCUSSION

Due to extensive drug resistance and adverse drug reactions, it is often difficult for patients with XDR-TB to obtain drugs for effective treatment. This study, which characterized the accumulation and dynamic changes of DRMs in three XDR-TB patients during 3−5 years of ineffective treatment, showed that in-host microevolution can be complex. New drug-resistant subpopulations can arise not only during treatment, but also after the patient is no longer receiving the drug. In other instances, the MTB resistance profile can remain stable for long periods without the emergence of new resistance mutations.

The acquisition of new drug-resistant subpopulations during the drug treatment is well known [20–22] but, surprisingly, we also observed new resistant subpopulations emerging after the drug had been stopped. There are two possible explanations for this phenomenon. One possibility is that after stopping slowly cleared drugs, such as BDQ, which has a half-life >5 months [23], the drug remains in the body at high concentrations for long periods, providing the selection pressure for the emergence of new drug-resistant subpopulations. For example, the subpopulation carrying the *atpE* E61D mutation appeared in patient A after BDQ was stopped. Another explanation is lesion heterogeneity. Multiple, spatially separated lesions can exist within the patient, with each lesion having its own microenvironment and its own MTB population that evolves independent of the MTB populations in other lesions [24]. This model proposes that the different resistant subpopulations found in sputum samples may have come from different lesions. For example, after patient A had stopped taking PAS for 7 months, a previously fixed *folC* mutation was completely replaced by a newly arising *thyA* mutation. Interestingly, a CT of patient A on 7 September 2023, when the subpopulation with *folC* mutation was dominant, showed the presence of a lesion in the lower lobe of the left lung. However, a subsequent CT study on 12 December 2023 showed resorption of the left lower lobe lesion and the appearance of a new cavity in the upper lobe of the right lung. Concomitantly, the *folC* mutant subpopulation had disappeared and was replaced by a new subpopulation with a large *thyA* deletion, suggesting that the two subpopulations may have been present in different lesions.

It appears that the genetic diversity of MTBs within the host is higher under ineffective treatment than during effective treatment [19]. It was, therefore, expected that new resistant subpopulations would arise in the three patients during their long-term ineffective treatments. What was unexpected, however, was the absence of new drug-resistant populations emerging during 9−48 months of ineffective treatment, perhaps because of insufficient drug concentrations within the lesions. The MTB populations in patients B and C remained susceptible to CS despite long-term treatment with the drug. This could be due to low concentrations of CS, which have been found in more than half of patients receiving it [25, 26]. Similarly, Eldholm *et al*. [11] reported that patients remained susceptible to PZA despite receiving it for 42 months, presumably due to pharmacokinetic factors that prevented the PZA from reaching effective concentrations within the patient [27]. The three patients we studied all had lung cavities, which can be difficult for drugs to penetrate sufficiently to reach therapeutic concentrations [28]. The patients continued to have positive sputum cultures despite long-term treatment with drugs that should have been effective, further suggesting that the drug concentrations within the lesions may have been inadequate to clear the bacteria. This “pseudo-therapy” phenomenon, in which the MTB is not drug resistant but the concentration of the drugs in the patient are subtherapeutic, can lead to treatment regimens that appear appropriate but in reality, are not effective.

*In vivo*, drug-resistant subpopulations are in a competitive environment, as reflected in subpopulation emergence, growth and turnover. Under long-term BDQ treatment, we found that patient A had multiple BDQ-resistant subpopulations carrying *mmpR* and/or *aptE* mutations and that the subpopulations competed with each other, eventually leaving only bacteria with the *mmpR* D47fs. This dynamic turnover of the *mmpR* and *aptE* mutant subpopulations has been reported previously in BDQ-treated patients [29] and attributed to a combination of drug selection pressure [8] and the relative fitness costs of DRMs [30]. Although mutations in both *mmpR* and *atpE* can confer resistance to BDQ [31], *atpE* is the target of BDQ [32] and *atpE* mutations generally confer higher levels of BDQ resistance [33]. In patient A, subpopulations with mutations in *aptE* emerged after subpopulations with mutations in the *mmpR* gene, perhaps leading to a higher level of resistance. However, the subpopulations with *atpE* mutations were eventually lost after BDQ was stopped, perhaps because AtpE is involved in ATP synthesis, and therefore *atpE* mutations may compromise bacterial fitness [34] while not providing any advantage in environments without BDQ. There is another possible explanation for the eventual dominance of *mmpR* D47fs subpopulation over the other subpopulations. We observed that this substitution process in patient A coincided with DLM resistance. Therefore, it is likely that DLM resistance occurred in the *mmpR* D47fs subpopulation but not in the other BDQ-resistant subpopulations. Under DLM pressure, the *mmpR* D47fs subpopulation with the acquired DLM resistance would have had a survival advantage that allowed it to eventually displace the other subpopulations.

In this study, both phenotypic and genotypic DSTs were used to detect drug resistance, and there were some instances of discordance between phenotypic and genotypic DST results. The phenomenon of phenotypic resistance but genetic sensitivity is not uncommon with BDQ, DLM, and CFZ, largely because the resistance mechanisms for these new/repurposed anti-TB drugs is incompletely understood and in many resistant strains the causal mutations have not yet been identified [18]. Phenotypic sensitivity but genotypic resistance was found in ETO and STR carrying the *ethA* D464fs (loss-of-function) and *gid* G73E mutations, respectively, which are classified as associated with resistance in the WHO catalogue [18]. These mutations may be associated with unreliable ETO phenotypic DST [35] and mutations in the *gid* gene are associated with low levels of STR resistance and MICs that may not reach the cutoff for defining resistance [36]. There may also be other potential factors, such as genetic background, that affect drug susceptibility [37, 38].

This study has some limitations. Only three patients and their serial isolates were included, making statistical analysis impossible. The generality of the phenomena we observed needs to be confirmed in studies of additional patients. Also, this study focused on bacterial factors, with limited information or assessment of the influence of host factors and patient pharmacokinetics on MTB microevolution.

## CONCLUSION

This study portrays the diversity of MTB microevolution *in vivo* in three patients with XDR-TB receiving ineffective treatment for 3−5 years. While some resistance profiles remained unchanged, we also observed the emergence of new resistant subpopulations and competition between resistant subpopulations both during treatment and after the drug was stopped. This study illustrates the diversity of MTB microevolution during prolonged, ineffective treatment of XDR-TB.

## Supplementary Material

Supplemental_figures_5_21_eoaf014

TableS1_eoaf014

## Data Availability

WGS data of MTB strains are stored at the National Genomics Data Center of China (https://ngdc.cncb.ac.cn/; BioProject accession: PRJCA040468).
