# Supplementary material for: Within-host population dynamics of extensively drug-resistant Mycobacterium tuberculosis revealed by an over 3-year longitudinal study
Source: Evol Med Public Health. 2025 Jul 1;13(1):167–75. doi: 10.1093/emph/eoaf014 (PMC12409779; doi:10.1093/emph/eoaf014)
Supplement: Supplemental_figures_5_21_eoaf014 [file supplemental_figures_5_21_eoaf014.docx]

**Supplementary figures for:**

**Heterogeneous within-host population dynamics of extensively drug-resistant *Mycobacterium tuberculosis* revealed by an over three-year longitudinal study**

**Supplementary figures**

**
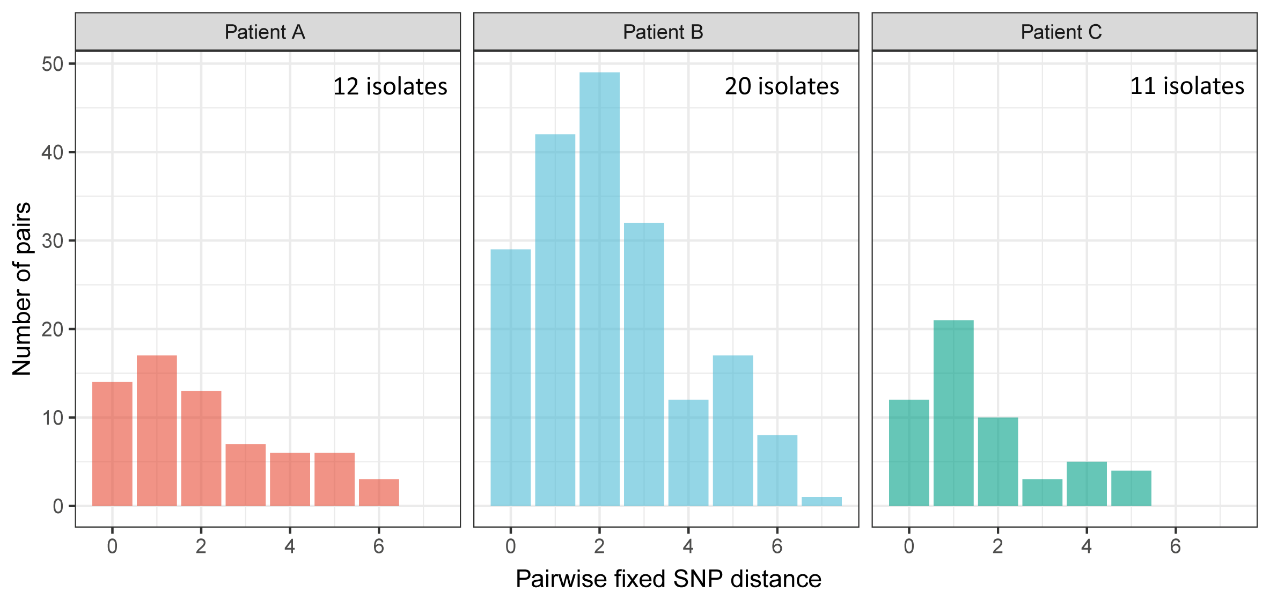
Supplementary Figure 1.** Histogram of fixed SNPs differences between serial patient isolates.

**
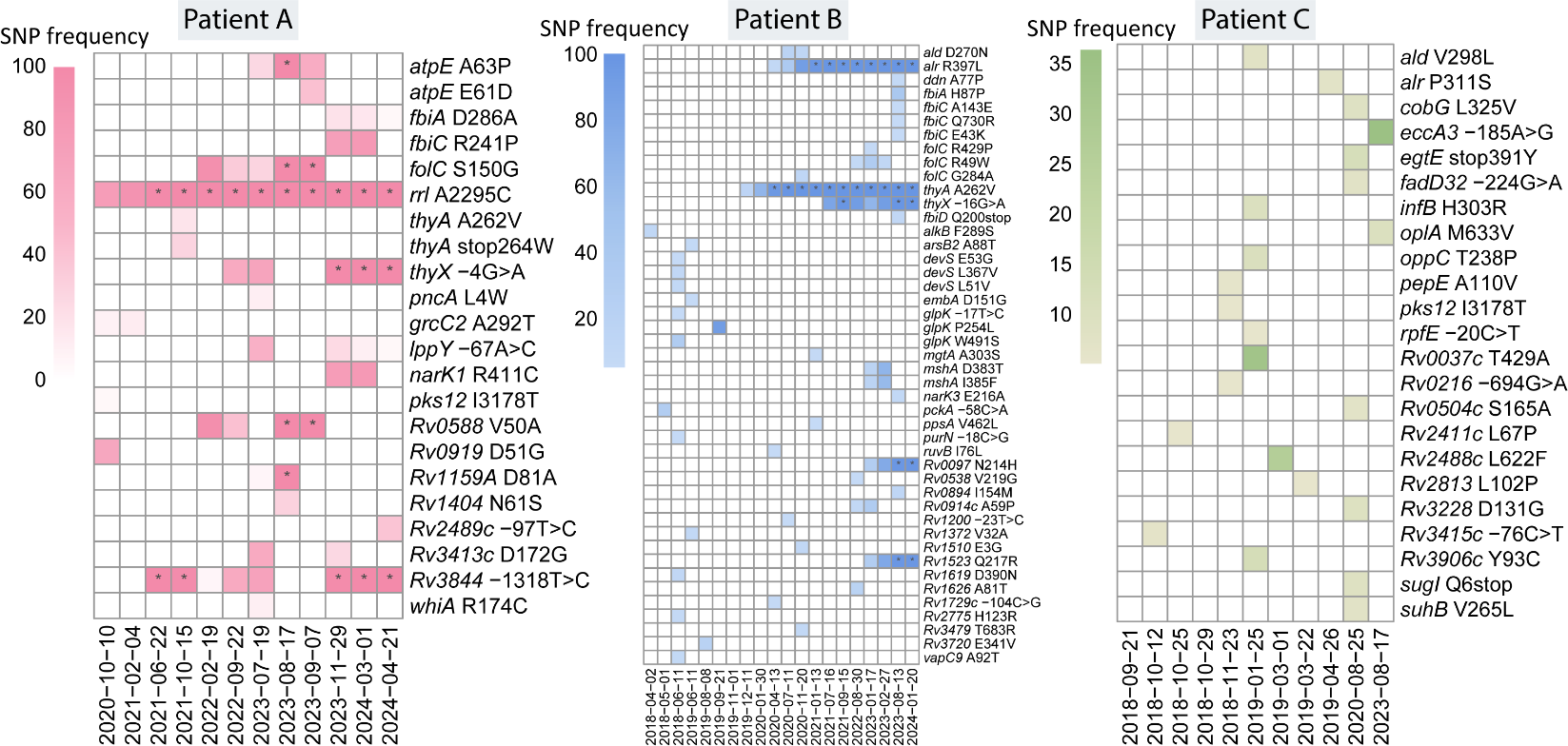
Supplementary Figure 2.** Heatmap of the frequencies of unfixed SNPs among serial isolates from patients A, B and C. The horizontal axis shows the patient isolates and the vertical axis shows the specific SNPs. Darker colors indicate higher frequencies of unfixed SNPs, and the asterisk (*) indicates that the SNP had become fixed.
